# RAS MAPK inhibitors deregulation in leukemia

**DOI:** 10.18632/oncoscience.274

**Published:** 2015-12-04

**Authors:** Eric Pasmant, Dominique Vidaud, Paola Ballerini

**Affiliations:** Laboratory of Hematology, Trousseau Hospital, AP-HP, Paris, France

**Keywords:** RAS-MAPK, SPRED1, NF1, acute myeloid leukemia, AML

The RAS (mitogen-activated protein kinase) MAPK pathway is commonly deregulated in human cancer, including childhood acute myeloid leukemias (AMLs). Canonical strong activating somatic mutation of RAS-MAPK pathway genes are recurrently found in AMLs. They affect integral components of the pathway, and upstream activators, and include NRAS, KRAS, BRAF, PTPN11, and FMS-related tyrosine kinase 3 (FLT3). Irrespective of etiology, the oncogenic RAS signal is frequently potentiated as leukemia progress; unknown mechanisms may contribute to this emerging aspect of leukemia evolution. It is well established that RAS activation can trigger compensatory feedback mechanisms that dampen signaling output. Suppression of these negative regulators may result in an enhancement of RAS signaling driving AML progression. Recent mathematical modeling and subsequent experiments revealed that mutations of the tumor suppressor gene *NF1* (*neurofibromin 1*) can amplify the effects of other RAS pathway mutations, including weakly activating, non canonical RAS mutants. Combinations of RAS-MAPK pathway mutations (including mutations of RAS-MAPK regulators) could therefore serve the role of leukemia driver [1].

SPRED1 and SPRED2 are members of the evolutionarily conserved SPROUTY/SPRED family of membrane-associated negative regulators of the RAS MAPK pathway. SPRED1 can interact with neurofibromin (the *NF1* gene product) by inducing the plasma membrane localization of the latter and the subsequent down-regulation of RAS-GTP levels [2]. Recently, we looked for *SPRED1* mutations in 230 cases of pediatric acute leukemia. We found 2% of patients carrying *SPRED1* germline mutations coexpressed with known lesions activating the RAS-MAPK pathway. Strikingly, *SPRED1* transcription was profoundly decreased in almost the totality of AMLs with consequent downregulation at the protein level. SPRED1 lowest levels were observed in FLT3-ITD mutated leukemia. Our study revealed a new general mechanism which contributes to deregulate RAS-MAPK pathway in pediatric AMLs [3].

Sprouty 4 (SPRY4), another RAS-MAPK inhibitor, was recently functionally validated as a tumor suppressor in AMLs [4]. Zhao et al. showed that in mice, premalignant myeloid cells harboring a Kras G12D allele, retained low levels of RAS signaling owing to negative feedback involving Spry4 that prevented transformation. *SPRY4* is located on human chromosome 5q, a region affected by large heterozygous deletions that are associated with aggressive disease in which gain-of-function mutations in the RAS pathway are rare. AML cells harbor losses of multiple RAS signaling negative regulatory genes that can functionally cooperate to achieve high levels of RAS pathway activation. Acquisition of RAS-MAPK pathway activation in certain AML subtype may therefore be driven by loss of negative regulators, like SPRED or SPRY proteins. As the level of RAS signaling output is critical for the transforming processes, concomitant loss of several of these genes may be needed to overcome the critical threshold of RAS activation. The loss of inhibitory proteins may indeed represent a decisive step to escape oncogene induced senescence triggered by RAS activating lesions and promote full cellular malignancy [4–5]. Given the number of potential negative regulators in this pathway, the foreseeable combinations that may replace oncogenic RAS (or others RAS activators) are seemingly very high. It is tempting to speculate that emerging actors, like DUSP (Dual Specific Phosphatases) or RAS-GAP (RAS GTPase activating proteins) genes might also be involved in RAS oncogenic signaling in AMLs.

Constitutional loss-of-function mutations in the *SPRED1* gene cause a rare phenotype referred as neurofibromatosis type 1 (NF1)-like syndrome or Legius syndrome, consisting in multiple café-au-lait macules, axillary freckling, learning disabilities, and macrocephaly. NF1 and Legius syndrome belong to the neuro-cardio-facio-cutaneous syndromes that are caused by deregulating constitutional mutations of the RAS-MAPK signaling pathway [2]. These RASopathies that include Noonan syndrome, LEOPARD syndrome, cardio-facio-cutaneous syndrome, Costello syndrome, NF1 and the Legius syndrome, share characteristic overlapping features, including predisposition to develop multiple types of cancer. Individuals with NF1 and Noonan syndrome have a higher risk of haematological malignancies, including acute leukemia and the rare disorder juvenile myelomonocytic leukemia. Although rare, inherited predispositions to myeloid leukemia have uncovered a critical role of hyperactive RAS-MAPK signaling in normal myeloid growth and leukemogenesis. We previously reported the observation of an 11-month-old boy with a *SPRED1* constitutional mutation, who developed an AML [6].

RAS mutations account for less than 10–15 % of pediatric AML and FLT3-ITD mutations do not exceed 5–8 %, which is in contrast with the higher frequency of MAPK/PI3K up-regulation observed in these leukemia. It is reasonable to think that negative-feedback regulators for RAS signaling are involved with AML transformation at genetic or epigenetic level [7]. Future studies are needed to integrate both somatic and germline variants in RAS-MAPK regulators, to provide comprehensive characterization of genetic risk factors for AML and shed light on the functional consequences of these alterations. This latter point is of utmost importance to develop more rational therapies aimed to inhibit the complex network of RAS-MAPK aberrant activation.
